# Suppression of Akt-mTOR pathway rescued the social behavior in *Cntnap2*-deficient mice

**DOI:** 10.1038/s41598-019-39434-5

**Published:** 2019-02-28

**Authors:** Xiaoliang Xing, Jing Zhang, Kunyang Wu, Beibei Cao, Xianfeng Li, Fang Jiang, Zhengmao Hu, Kun Xia, Jia-Da Li

**Affiliations:** 1 Center for Medical Genetics, School of Life Sciences, Central South University, Changsha, 410078 Hunan P. R. China; 2 Hunan University of Medicine, Huaihua, 418000 Hunan P. R. China; 3 Hunan Key Laboratory of Animal Models for Human Diseases, Changsha, 410078 Hunan P. R. China; 4 Hunan Key Laboratory of Medical Genetics, Changsha, 410078 Hunan P. R. China; 5 Collaborative Innovation Center for Genetics and Development, Shanghai, 200000 China

## Abstract

Autism spectrum disorders (ASD) form a heterogeneous, neurodevelopmental syndrome characterized by deficits in social interactions and repetitive behavior/restricted interests. Dysregulation of mTOR signaling has been implicated in the pathogenesis of certain types of ASD, and inhibition of mTOR by rapamycin has been demonstrated to be an effective therapeutics for impaired social interaction in *Tsc1*+/*−*, Tsc2+/*−*, *Pten*−/− mice and valproic acid-induced ASD animal models. However, it is still unknown if dysregulation of mTOR signaling is responsible for the ASD-related deficit caused by other genes mutations. *Contactin associated protein-like 2* (*CNTNAP2*) is the first widely replicated autism-predisposition gene. Mice deficient in *Cntnap2* (*Cntnap2*−/− mice) show core ASD-like phenotypes, and have been demonstrated as a validated model for ASD-relevant drug discovery. In this study, we found hyperactive Akt-mTOR signaling in the hippocampus of *Cntnap2*−/− mice with RNA sequencing followed with biochemical analysis. Treatment with Akt inhibitor LY294002 or mTOR inhibitor rapamycin rescued the social deficit, but had no effect on hyperactivity and repetitive behavior/restricted behavior in *Cntnap2*−/− mice. We further showed that the effect of LY294002 and rapamycin on social behaviors is reversible. Our results thus identified hyperactive Akt-mTOR signaling pathway as a therapeutic target for abnormal social behavior in patients with dysfunction of CNTNAP2.

## Introduction

Autism spectrum disorders (ASD) are a complex set of heterogeneous neurodevelopmental disorders characterized by core deficits in social behavior and communication, accompanied by restricted interests and repetitive behaviors, affecting up to 1 in 68 children^1,2^. ASD brings a substantial economic burden, including high health-care and school costs and loss of income for caregivers^3^. However, current available pharmaceutics for ASD is limited to the treatment of co-occurring behaviors or diagnoses, instead of ASD *per se*^4^.

Accumulative evidences suggest the important roles of Akt-mTOR signaling in the pathogenesis of ASD^4–7^. First, abnormal mTOR signaling has been observed in several transgenic ASD animal models, such as *Tsc1*+/*−*, *Tsc2*+/*−*, *Pten*−/− and *Fmr1*−/− mice^4,5^. Second, environmental factors, such as valproic acid, that elicit ASD may also affect the mTOR signaling^6^. Finally, the inhibitor of mTOR signaling, rapamycin, has been demonstrated to be an effective therapeutics for impaired social interaction in *Tsc1*+/*−*, *Tsc2*+/*−*, *Pten*−/− mice and valproic acid-induced ASD animal model^4,8,9^. However, ASD are highly heritable, and genetic studies have revealed extraordinary heterogeneity with hundreds of rare risk genes, none accounting for more than 1% of ASD instances^10–12^. It is still unknown if dysregulation of mTOR signaling is responsible for the ASD-related deficit caused by other gene mutations.

*Contactin associated protein-like 2* (*CNTNAP2*) is the first widely replicated autism-predisposition gene^13–16^. Knockdown of *Cntnap2* produces a cell-autonomous decrease in dendritic arborization and spine development in pyramidal neurons, leading to a global decline in excitatory and inhibitory synapse numbers and a decrease in synaptic transmission^17^. Mature neurons from *Cntnap2*−/− mice show reduced spine density and decreased level of GluA1 subunits of AMPA receptors in spines^18^. *Cntnap2*−/− mice show core ASD-like phenotypes, including impaired social behaviors and repetitive behavior^19^.

*Cntnap2*−/− mice have been demonstrated as a validated model for ASD-relevant drug discovery. Risperidone, a FDA-approved drug for symptomatic treatment of ASD, reduces hyperactivity and perseveration in *Cntnap2*−/− mice, but has no effect on social deficit^19^. Neuropeptide oxytocin, involved in the modulation of various aspects of social behaviors, restores the impaired social behavior of *Cntnap2*−/− mice^20^. Furthermore, real-time modulation of balance between neuronal excitation and inhibition also rescues social behavior deficit of *Cntnap2*−/− mice^21^. Recently, Kim *et al*. demonstrated that the positive-allosteric-modulator for AMPA receptor is able to rescue the social deficit in *Cntnap2*−/− mice^22^.

In this study, we found hyperactive Akt-mTOR signaling in the hippocampus of *Cntnap2*−/− mice with RNA sequencing followed with biochemical analysis. Pharmacological inhibition of Akt-mTOR signaling reversibly rescued social deficit, but had no effect on hyperactivity and repetitive behaviors in *Cntnap2*−/− mice.

## Materials and Methods

### Animals

*Cntnap2*+/*−* mice were obtained from the Jackson Laboratory (#017482). *Cntnap2*−/− mice and wild-type (WT) mice were obtained from heterozygous crossing and were born with the expected Mendelian frequencies. The genotype was confirmed by PCR. Mice were group-housed with 4–6 mice per cage, in a room on a 12 h light/12 h dark cycle (lights on at 5:00 AM, off at 5:00 PM) maintained at 22 ± 2 °C. All procedures regarding the care and use of animals are in accordance with the Institutional Guidelines. PCR primers were listed as followed:

Forward primer: CTGCCAGCCCAGAACTGG;

WT reverse primer: CGCTTCCTCGTGCTTTACGGTAT;

Mutant reverse primer: ACACCAGGGGCAAGAATTG.

All animal experiments were approved by the ethics committee of Center for Medical Genetics, School of Life Sciences, Central South University of China. All methods were performed in accordance with approved guidelines.

### Quantitative reverse transcription–PCR

Total RNA from the hippocampus of male WT and Cntnap2−/− mice was extracted using Trizol reagent (Life technologies, NY, USA) according to the manufacturer’s instruction. 2.0 μg of total RNA were reverse-transcribed using the RevertAid First Strand cDNA Synthesis Kit (Thermo Fisher, Waltham mass, USA). The mRNA levels were examined with qPCR using 1 × SYBR Green PCR master mix (Thermo Fisher, Waltham mass, USA) by a C1000 touch Thermal Cycler.

*GAPDH* forward primer: AGGTCGGTGTGAACGGATTTG

*GAPDH* reverse primer: TGTAGACCATGTAGTTGAGGTCA

*Met* forward primer: GTGAACATGAAGTATCAGCTCCC

*Met* reverse primer: TGTAGTTTGTGGCTCCGAGAT

*EfnA5* forward primer: AGCCAGGGTTGATGAGTAGAG

*EfnA5* reverse primer: GAACGTGGGTATCGGGGTG

### Western Blot

Mice at the age of 4–8 weeks without any behavioral test were sacrificed by cervical dislocation. The hippocampus and cortex from male WT and *Cntnap2*−/− mice were homogenized by a tissue homogenizer in 2 × SDS gel-loading buffer (50 mM Tris–HCl at pH 6.8, 2% SDS and 10% glycerol) with 1 × NaF, 1 × NaVO4, and 1 × Protein inhibitor cocktail. The supernatant was obtained by centrifugation, and the protein concentration was determined using the PierceTM BCA protein Assay kit (Thermo Fisher, Waltham mass, USA). Proteins were resolved by SDS–PAGE, transferred onto a polyvinylidene fluoride membrane and blocked in 5% skim milk/Tris-buffered saline that contained 0.1% Tween 20 at room temperature for 1 h. The membranes were incubated with the primary antibodies at 4°C overnight, and then were incubated with second antibody at room temperature for 1 h. After washing, the bands were visualized with Enhanced chemiluminescence Western blotting detection reagents. The band density was quantified using ImageJ software. The antibodies were listed as following: Cntnap2 (ab33994, Abcam, USA), Phospho-Akt (Ser473) (4060, CST, USA), Akt (4691, CST, USA), Phospho-mTOR (Ser2448) (5536, CST, USA), mTOR (2983, CST, USA), Phospho-S6 (Ser235/236) (2211, CST, USA), S6 (2217, CST, USA), β-actin (A2228, Sigma, USA).

### Drug administration

LY294002 (HY-10108) or rapamycin (HY-10219) were obtained from MedChem Express (MCE, New Jersey, USA). Male mice at the age of 4–8 weeks were received LY294002 (25 mg/kg body weight)^23,24^ and rapamycin (10 mg/kg body weight)^25^ or an equal volume of vehicle by intraperitoneal injection (i.p.) once/day for 2 consecutive days. The behavioral tests or tissue collection were performed at 60 min after the second administration. Each mouse only received injection of one drug (saline, LY294002 or rapamycin).

### Social approach (three-chamber) test

The social approach test was performed as described^26,27^. The apparatus consisted of three Plexiglas chambers: the central chamber and two side chambers. Each chamber was accessible to the mouse from the center through the retractable doorways. The test consisted of two phases: habituation and sociability.

At phase 1, two empty plexiglas cages were placed in two side chambers, and a test mouse was placed in the central chamber and was given the choice to explore all three chambers for 10 min.

At phase 2, a stranger mouse (matched in age and sex, stranger 1), which was enclosed in a plexiglas cage to make sure that only the test mouse could initiate social interaction, was placed into cage 1 for replaced for object 1. The test mouse was placed in the central chamber and was allowed to explore the three chamber apparatus for 10 min to assess the sociability (interaction with stranger 1).

The behaviors were recorded and the sniffing time of the test mouse for each plexiglas cage were scored by Anilab Software (Anilab, Ningbo, China). The preference index was calculated as showed below:

$${\rm{Preference}}\,{\rm{index}}=\frac{{\rm{Time}}\,{\rm{exploring}}\,({\rm{stranger}}\,{\rm{mouse}})-{\rm{Time}}\,{\rm{exploring}}\,({\rm{object}})}{{\rm{Time}}\,{\rm{exploring}}\,({\rm{stranger}}\,{\rm{mouse}})+{\rm{Time}}\,{\rm{exploring}}\,({\rm{object}})}$$

### Reciprocal social interaction

Mice were placed in a cage to which they had been previously habituated (for 10 min) with an unfamiliar mouse matched in age, genotype, and sex for 10 min. Both mice in the pair were treated either with the same drug or with vehicle. The time engaged in social interaction (nose-to-nose sniffing, nose-to-anus sniffing, and following or crawling on/under each other) for the pair (combining the behavior of both animals) was measured by two independent human observers^5^.

### Open field test

Mice were individually placed in a Plexiglas box (62 cm × 62 cm). The distance travelled was recorded for 10 min and scored by Anilab software (Anilab, Ningbo, China).

### Grooming test

Mice were individually placed in a Plexiglas column (20 cm diameter). After acclimation for 10 minutes, the behaviors were recorded for 10 minutes. The time spent for self-grooming was measured by a researcher blind to the genotypes.

### Statistical analysis

A repeated-measure ANOVA followed by Bonferroni *post hoc* tests or unpaired two-tail Student’s *t* test was used as indicated. All statistical analyses were performed using the Prism 6.01 (Graph Pad Software, San Diego, CA).

## Results

### *Cntnap2*-deficiency led to hyperactivity in the Akt-mTOR signaling pathway

**Figure 1:**
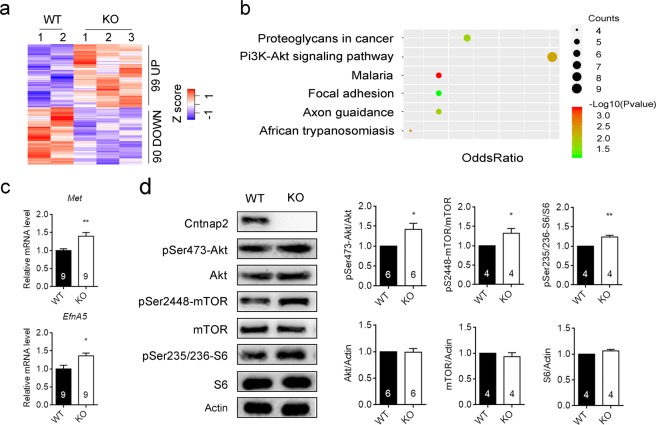
*Cntnap2*-deficiency affected genes expression and led to hyperactivity in the PI3K-Akt signaling. (**a**) Gene expression in the hippocampus from WT and *Cntnap2*−/− mice as assayed with RNA-seq. (**b**) The differentially expressed genes were enriched in 6 different signaling pathways as analyzed with KEGG. (**c**) The expression of *Met* and *EfnA5* were increased significantly in the hippocampus of *Cntnap2*−/− mice as assayed with qPCR. (**d**) Representative immunoblots and quantification of lysates from the hippocampus of WT and *Cntnap2*−/− mice. The phosphorylation levels of Akt (Ser473), mTOR (Ser2448) and S6 (Ser235/236) were significantly increased in the hippocampus of *Cntnap2*−/− mice. The number of mice was indicated in the respective graphs. *p < 0.05, **p < 0.01. Data were expressed as the mean ± sem (standard error of the mean), unpaired two-tail Student’s *t* test. Raw data for panel a was provided in Supplementary Table 1a. Raw gel images for panel d was provided in Supplementary Fig. 1d.

ASD may be associated with dysfunction in a variety of brain areas, such as the hippocampus^28^, the prefrontal cortex^21^, and striatum^29^. To identify potential therapeutic targets for *Cntnap2*−/− mice, we analyzed the global gene expression in the hippocampus from wild-type (WT) and *Cntnap2*−/− mice by RNA-sequencing (RNA-seq). We identified 90 significantly down-regulated genes (ratio <0.8), and 99 significantly up-regulated genes (ratio >1.2) in *Cntnap2*−/− mice (Fig. 1a, Supplementary Table 1a). All significantly changed genes were enriched in 6 different signaling pathways as analyzed with Kyoto Encyclopedia of Genes and Genomes (KEGG) (Fig. 1b). Notably, there were 9 genes enriched in the phosphatidylinositol 3-kinase (PI3K)-Akt signaling pathway (Supplementary Table 1a). The expression of *Met proto-oncogene receptor* (*Met*) and *EphrinA5 (EfnA5*) were further verified by quantitative reverse transcription PCR (qRT-PCR) (Fig. 1c).

The up-regulation of *Met* and *EfnA5* suggested a hyperactive PI3K-Akt signaling in the *Cntnap2*−/− mice. We then examined the activation of the PI3K-Akt signaling pathway by immnoblotting analysis on the Akt phosphorylation level. As shown in Fig. 1d, the phosphorylation level of Akt (Ser473) was increased significantly in *Cntnap2*−/− mice. In contrast, the level of total Akt was not changed significantly. The mTOR signaling pathway is a typical downstream of Akt signaling. The phosphorylation levels of mTOR (Ser2448) and its downstream target molecule ribosomal protein S6 (S6) (Ser235/236) were also increased significantly in *Cntnap2*−/− mice, whereas the levels of total mTOR or S6 were not significantly changed (Fig. 1d).

The hyperactive Akt-mTOR signaling seems to be a common consequences of *Cntnap2* deficiency as it was also detected in the cortex and dorsal root ganglion neurons from *Cntnap2*−/− mice (Supplementary Fig. 1d, and data not shown).

### Inhibition of Akt-mTOR signaling rescued social deficit in *Cntnap2*−/− mice

**Figure 2:**
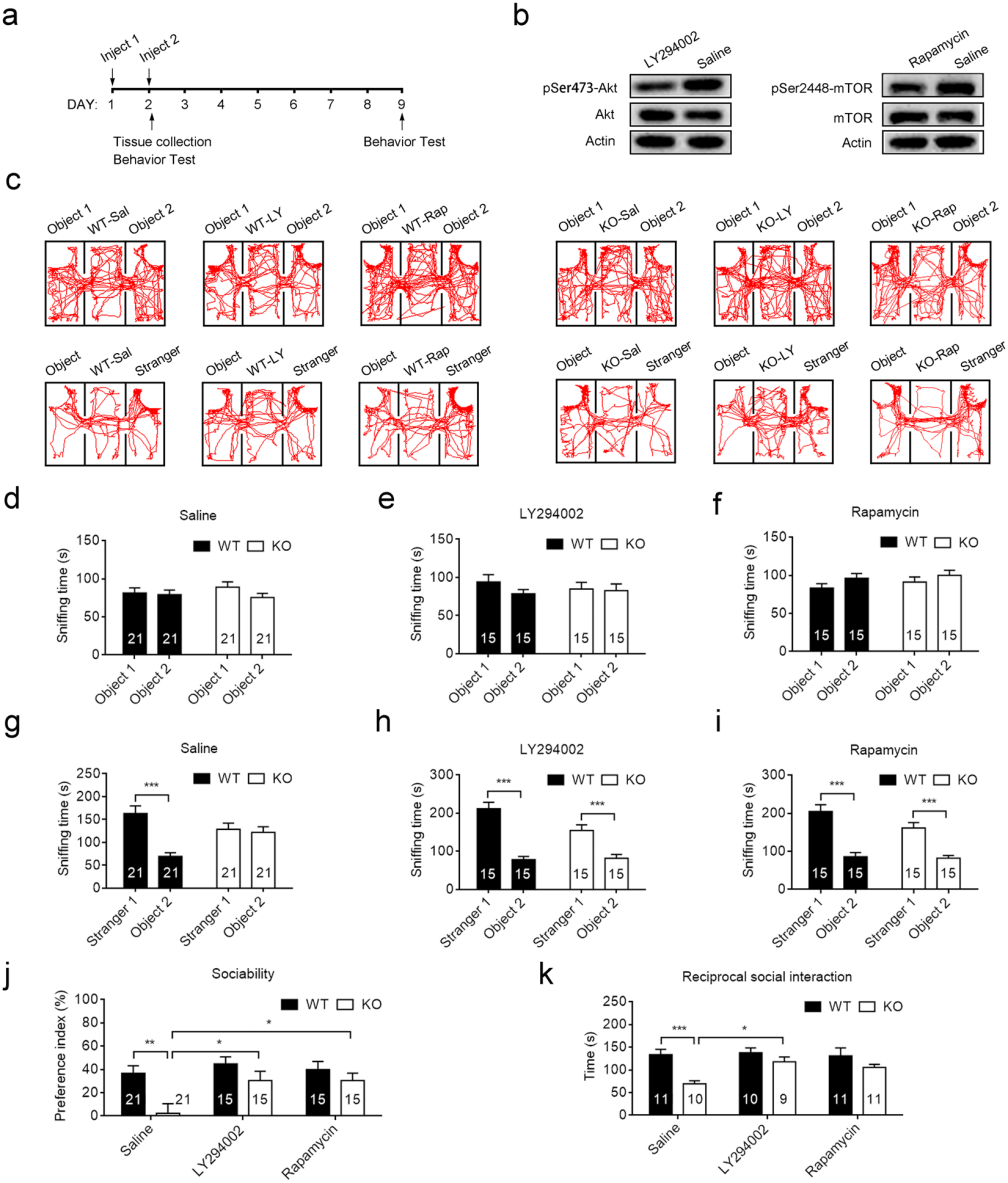
Inhibition of Akt/mTOR signaling rescued social deficit in *Cntnap2*−/− mice. (**a**) The schematic diagram of drug administration and western blot or behavior test. (**b**) Representative immunoblots of lysates from the hippocampus of WT mice treated with LY2094002 or rapamycin at 60 min after the second injection. (**c**) Representative exploratory activities in the three-chamber test of WT and *Cntnap2*−/− mice with various treatments. (**d**–**f**) The time of mice spent on sniffing in the habituation phase after i.p. injection of saline (**d**) or LY294002 (**e**) or rapamycin (**f**) for two consecutive days. Unpaired two-tail Student’s *t* test. *p < 0.05. Data were expressed as the mean ± sem. (**g**–**i**) The time of mice spent on sniffing in the sociability phase after i.p. injection of saline (**d**) or LY294002 (**e**) or rapamycin (**f**) for two consecutive days. Unpaired two-tail Student’s *t* test. *p < 0.05, **p < 0.01, ***p < 0.001. Data were expressed as the mean ± sem. (**j**) The preference index of WT and Cntnap2−/− mice treated with saline, LY294002 or rapamycin. A repeated-measure ANOVA followed by Bonferroni *post hoc* tests. (**k**) The reciprocal social interaction time WT and Cntnap2−/− mice treated with saline, LY294002 or rapamycin. A repeated-measure ANOVA followed by Bonferroni *post hoc* tests. *p < 0.05, **p < 0.01, ***p < 0.001. Data were expressed as the mean ± sem. The number of mice was indicated in the respective graphs. Raw gel images for panel b was provided in Supplementary Fig. 2b.

To see whether hyperactive PI3K-Akt signaling was responsible for the social deficit in *Cntnap2*−/− mice, we sought to analyze the consequences of inhibition of PI3K-Akt signaling by Akt inhibitor LY294002 (Fig. 2a). We used the three-chamber test to assess the sociability. In the habituation phase, WT and *Cntnap2*−/− mice showed no difference in the sniffing time (Fig. 2c,d). Consistent with previous reports, *Cntnap2*−/− mice showed significantly reduced interaction for exploring a stranger mouse relative to an inanimate object (Fig. 2c,g)^19^.

Male mice at the age of 4–8 weeks received LY294002 (25 mg/kg body weight) or an equal volume of vehicle once/day for 2 consecutive days. The behavioral tests or tissue collection were performed at 60 min after the second administration (Fig. 2a). Treatment with LY294002 significantly suppressed the phosphorylation of Akt (Fig. 2b). LY294002 treatment did not alter interaction time in habituation phase (Fig. 2c,e), but significantly improved the social interaction of *Cntnap2*−/− mice. As shown in Fig. 2h, LY294002-treated *Cntnap2*−/− mice spent significantly more time with a stranger mouse than the object. The preference index of saline-treated *Cntnap2*−/− mice was significantly lower than that of saline-treated WT mice (Fig. 2j); however, the preference index of *Cntnap2*−/− mice was significantly increased by LY294002 treatment to the levels comparable with WT mice (Fig. 2j).

The FDA-approved drug, rapamycin, showed beneficial effect on the social behaviors in certain ASD animal models with over-activated mTOR signaling^4,5^. Recently, rapamycin was also revealed to improve the ASD-associated behaviors in patients with tuberous sclerosis^30^. As mTOR signaling was significantly increased in the *Cntnap2*−/− mice (Fig. 1d), we sought to determine the effect of rapamycin on the social behaviors. Consistent with previous study, administration of rapamycin significantly suppressed the phosphorylation of S6 (Fig. 2b). Rapamycin did not alter interaction time in habituation phase (Fig. 2c,f), but significantly improved the social interaction of *Cntnap2*−/− mice. Rapamycin-treated *Cntnap2*−/− mice spent significantly more time with a stranger mouse than the object (Fig. 2c,i). As a result, the preference index of rapamycin-treated *Cntnap2*−/− mice was significantly increased to the levels comparable with WT mice (Fig. 2j).

We also measured the therapeutic effect of LY294002 and rapamycin on social deficit of *Cntnap2*−/− mice using a reciprocal social interaction test. As shown in Fig. 2k, saline-treated *Cntnap2*−/− mice spent significantly less time on reciprocal social interaction than the saline-treated WT mice (Fig. 2k). However, LY294002 or rapamycin treatment increased the reciprocal social interaction time to the similar levels of WT mice (Fig. 2k).

### The rescuing effect of Akt-mTOR inhibitors on the social behavior was reversible

**Figure 3:**
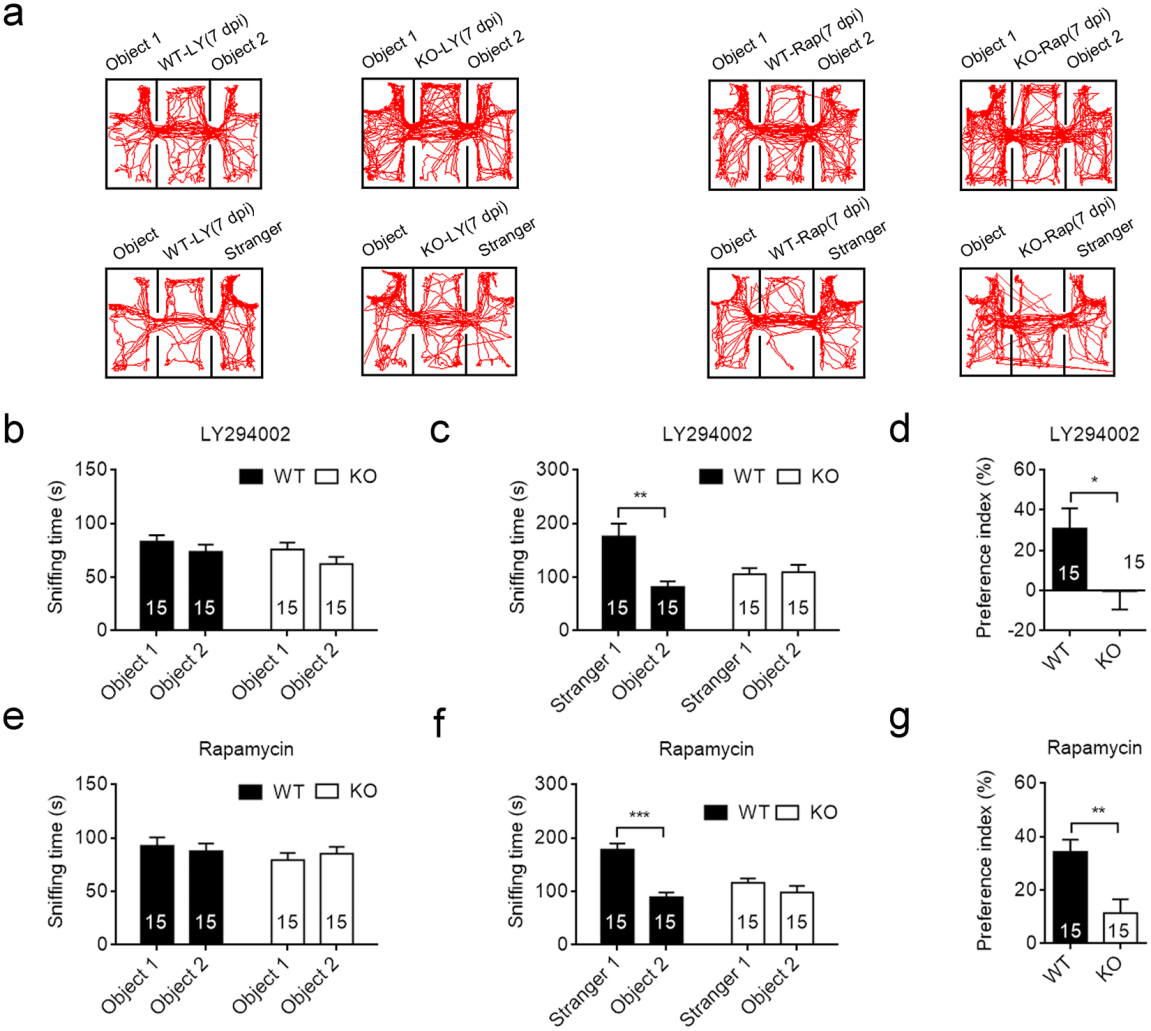
The rescuing effect of Akt/mTOR inhibitors for *Cntnap2*−/− mice was reversible. (**a**) Representative exploratory activities in the three-chamber test of WT and *Cntnap2*−/− mice after 7 days of last injection. (**b**,**c**) The duration time of mice spent on sniffing with a strange mouse or an object at 7 days post injection of LY294002. (**d**) The social preference index of WT and *Cntnap2*−/− mice at 7 days post injection of LY294002. (**e**,**f**) The duration time of mice spent on sniffing with a strange mouse or an object at 7 days post injection of rapamycin. (**g**) The social preference index of WT and Cntnap2−/− mice at 7 days post injection of rapamycin. The number of mice was indicated in the respective graphs. *p < 0.05, **p < 0.01, ***p < 0.001. Data were expressed as the mean ± sem, unpaired two-tailed student’s *t* test. d.p.i., days post injection.

To see if LY294002 and rapamycin treatment had long-lasting effect on the social behavior, we measured the sociability with the three-chamber test in drug-treated mice at 7 days post of injection. The drug-treated WT and *Cntnap2*−/− mice spent comparable time sniffing the two objects (Fig. 3a,b,e). However the drug-treated *Cntnap2*−/− mice could not distinguish the stranger mouse and the inanimate object (Fig. 3a,c,f). The preference index for drug-treated *Cntnap2*−/− mice was significantly lower than that of drug-treated WT mice at 7 days after last injection (Fig. 3d,g). Our results therefore indicated that Akt inhibitor LY294002 or mTOR inhibitor rapamycin only had a transient effect on the social deficit in *Cntnap2*−/− mice.

### Inhibition of Akt-mTOR signaling had no effect on the hyperactivity and repetitive behaviors in *Cntnap2*−/− mice

**Figure 4:**
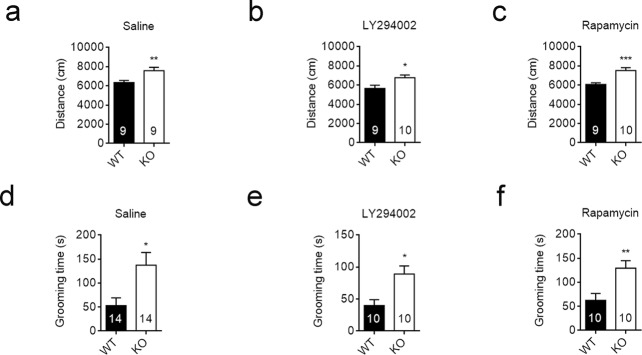
Inhibition of Akt/mTOR signaling had no effect on the hyperactivity and repetitive behaviors in *Cntnap2*−/− mice. (**a**–**c**) The distance travelled in 10 min in the open field by WT and Cntnap2−/− mice treated with saline (**a**), LY294002 (**b**) or rapamycin (**c**). (**d–f**) The time spent on self-grooming of WT and Cntnap2−/− mice treated with saline (**d**), LY294002 (**e**) or rapamycin (**f**). The number of mice was indicated in the respective graphs. *p < 0.05, **p < 0.01, ***p < 0.001. Data were expressed as the mean ± sem, unpaired two-tailed student’s *t* test.

Deficiency in *Cntnap2* also led to other autism-related behaviors, including hyperactivity and repetitive behaviors^19^. As shown in Fig. 4a, *Cntnap2*−/− mice displayed significantly higher locomotor activity than the WT controls in the open field test. Inhibition of Akt/mTOR signaling by LY294002 or rapamycin had no effect on the hyperactivity. The distance travelled by *Cntnap2*−/− was still higher than WT controls after drugs treatment (Fig. 4b,c).

Repetitive behavior is frequently observed in children with ASD^2^. We used a grooming test to measure the repetitive behavior of WT and *Cntnap2*−/− mice. Consistent with previous reports, *Cntnap2*−/− mice spent significantly more time on grooming themselves than the WT controls (Fig. 4d)^19^. Inhibition of Akt/mTOR signaling by LY294002 or rapamycin had no effect on the repetitive behaviors in *Cntnap2*−/− mice (Fig. 4e,f).

## Discussion

Dysregulation of mTOR signaling and synaptic pathology have been considered as two common denominators of ASD across different etiologies^31,32^. Many synaptic genes have been implicated in ASD, including *Cntnap2*, *Neuroligins* and *Shanks*^4,5,33^. Mice deficient in these synaptic genes display core autism-related deficits^19,26,34,35^. Over-activated mTOR signaling may elicit the ASD-like behaviors through increased synapse protein synthesis^4,36^. A rare single nucleotide polymorphism has been identified in autism that was associated with increased promoter activity in the *eukaryotic translation initiation factor* 4*E* (*eIF4E*) gene^37^. As a downstream molecule of mTOR signaling, eIF4E is also negatively regulated by 4E-binding proteins (4EBPs). Up-regulation of eIF4E in mice, either by eIF4E transgene or by 4EBP2-deletion, leads to ASD-like behaviors^38,39^. The *eIF4E* transgenic mice exaggerate translation and synaptic function, resulting in core ASD-like behaviors, and these ASD-like behaviors could be reversed by translation inhibitor 4EGI-1^39^. Interestingly, the eIF4E-dependent synaptic protein translations, such as *NLGN 1*/*2/3*/*4* are significantly increased in *4EBP2*−/− mice. Both pharmacological infusion of 4EGI-1 and knockdown of *NLGN1* could reverse the social deficits in *4EBP2*−/− mice^38^.

Intriguingly, synaptic impairments also affect the status of mTOR signaling, probably forming a molecular loop underlying the pathogenesis of ASD. Shank protein family is a group of synaptic scaffolding proteins. Mutations in *Shank2* and *Shank3* have been identified in individuals with ASD, and mice deficient in *Shank2* or *Shank3* showed core ASD-related deficits^26,34^. A recent study shows that *Shank3* deficiency leads to reduction in Akt-mTOR signaling, due to increased steady-state levels of Cdc2-like kinase 2 (CLK2). A CLK2 inhibitor successfully rescues the social deficit in *Shank3*−/− mice^26^. Puzzlingly, mTOR signaling is also significantly decreased in the transgenic mice overexpressing Shank3^40^.

In this study, we showed that deficiency in synaptic protein Cntnap2 also led to altered mTOR signaling, reinforcing the reciprocal relation between mTOR and synaptic signaling. The increased expression of *Met* and/or *EfnA5* may contribute partially to the hyperactive Akt-mTOR signaling. However, it is still unclear how *Cntnap2* deficiency causes up-regulation of *Met* and *EfnA5*. Anderson *et al*. reported that knockdown of *Cntnap2* leads to a global decline in synapse numbers and a decrease in synaptic transmission^17^, which may mediate the *Cntnap2*-deficiency and altered gene expression. Furthermore, the *Met* expression is also regulated epigenetically by methyl-CpG-binding protein 2 (MECP2)^41^. Deficiency in MECP2 resulted in Rett syndrome and ASD^42^. Interestingly, *EfnA5* is up-regulated nearly 40% in *MeCP2*−/− mice^43^, implying MeCP2 as potential mediator for *Cntnap2*-deficiency to the expression of *Met* and *EfnA5*.

Our pharmacological experiments indicated that hyperactive Akt-mTOR signaling is responsible for the social deficit, as inhibition of Akt or mTOR signaling reversibly rescued the social deficit in *Cntnap2*−/− mice. Nevertheless, treatment with Akt or mTOR inhibitors failed to normalize the hyperactivity and repetitive behaviors in *Cntnap2*−/− mice. Our data further supports the notion that distinct pathways lead to the social and repetitive behavioral deficits in ASD. Indeed, risperidone reduces hyperactivity, motor stereotypies and perseveration, but has no effect on the social behaviors in *Cntnap2*−/− mice^19^. Oxytocin treatment restores social behavior, but has no effect for repetitive behavior and hyperactivity^20^. Nevertheless, we cannot completely exclude the possibility that the persistence of hyperactivity and repetitive behaviors in the presence of LY294002 and rapamycin may be the results of insufficient inhibition of Akt-mTOR pathways in the brain regions mediating these behaviors. Indeed, Selimbeyoglu *et al*. demonstrated that modulation of prefrontal cortex excitation/inhibition balance is able to rescues social behavior as well as hyperactivity in *Cntnap2*−/− mice^21^. In summary, our data showed that deficiency in *Cntnap2* led to hyperactive Akt-mTOR signaling. Inhibition of Akt-mTOR signaling reversibly rescued the social deficit in *Cntnap2*−/− mice. Our study thus implied mTOR signaling as a common therapeutic target for ASD from different etiologies.

## Supplementary information

Supplementary Information

## Data Availability

The datasets generated and analyzed for the current study are available.
