# Supplementary material for: Suppression of Akt-mTOR pathway rescued the social behavior in Cntnap2-deficient mice
Source: Sci Rep. 2019 Feb 28;9:3041. doi: 10.1038/s41598-019-39434-5 (PMC6395585; doi:10.1038/s41598-019-39434-5)
Supplement: Supplementary file 1 — Supplementary Information [file 41598_2019_39434_MOESM1_ESM.pdf]

Suppression of Akt-mTOR pathway rescued the social behavior in *Cntnap2*-deficient mice

Xiaoliang Xing[1,2,3,4], Jing Zhang[1,3,4], Kunyang Wu[1,3,4], Beibei Cao[1,3,4], Xianfeng Li[1,3,4],

Fang Jiang[1,3,4], Zhengmao Hu[1,4,5], Kun Xia[1,4,5], Jia-Da Li[1,3,4,5*]

Supplementary table 1a

The list of 189 genes that were differentially expressed in the hippocampus of WT and *Cntnap2-/-* mice as analyzed by RNA-seq.

Gene	Ratio (KO/WT)	P Value	Gene Description
Cntnap2	0.16	0.000	contactin_associated_protein-like_2
Alas2	0.44	0.000	aminolevulinic_acid_synthase_2_erythroid
Hbb-bt	0.55	0.000	hemoglobin_beta_adult_t_chain
Ndn	0.58	0.000	necdin
Rpl38-ps2	0.58	0.012	ribosomal_protein_L38_pseudogene_2
Ikzf5	0.59	0.001	IKAROS_family_zinc_finger_5
**Col6a4**	**0.59**	**0.018**	**collagen_type_VI_alpha_4**
Gvin1	0.60	0.003	GTPase_very_large_interferon_inducible_1
Myo1f	0.61	0.003	myosin_IF
Hbb-bs	0.63	0.000	hemoglobin_beta_adult_s_chain
Klf2	0.63	0.003	Kruppel-like_factor_2_(lung)
Hba-a1	0.63	0.000	hemoglobin_alpha_adult_chain_1
Mki67	0.64	0.006	antigen_identified_by_monoclonal_antibody_Ki_67
Dynlt1b	0.64	0.005	dynein_light_chain_Tctex-type_1B
Hba-a2	0.64	0.000	hemoglobin_alpha_adult_chain_2
Gm27042	0.64	0.005	predicted_gene_27042
**Cdkn1a**	**0.65**	**0.007**	**cyclin-dependent_kinase_inhibitor_1A_(P21)**
Degs2	0.65	0.012	delta(4)-desaturase_sphingolipid_2
Gm42528	0.65	0.005	predicted_gene_42528

Gm3244	0.65	0.014	predicted_pseudogene_3244
Slfn5	0.65	0.009	schlafen_5
Gm42608	0.65	0.015	predicted_gene_42608
Tfap4	0.65	0.009	transcription_factor_AP4
Esam	0.66	0.001	endothelial_cell-specific_adhesion_molecule
Tmem181b-ps	0.67	0.000	transmembrane_protein_181B_pseudogene
Eng	0.67	0.000	endoglin
Chst9	0.67	0.016	carbohydrate_(N-acetylgalactosamine_4-0)_sulfotransferase_9
Zfp647	0.67	0.022	zinc_finger_protein_647
Btbd19	0.68	0.019	BTB_(POZ)_domain_containing_19
**Flt4**	**0.68**	**0.030**	**FMS-like_tyrosine_kinase_4**
Chrna5	0.68	0.007	cholinergic_receptor_nicotinic_alpha_polypeptide_5
Gm15751	0.68	0.030	predicted_gene_15751
Herc6	0.69	0.039	hect_domain_and_RLD_6
Filip1l	0.69	0.021	filamin_A_interacting_protein_1-like
Hspb1	0.69	0.035	heat_shock_protein_1
Ncf2	0.69	0.019	neutrophil_cytosolic_factor_2
Serpinf1	0.69	0.013	serine_(or_cysteine)_peptidase_inhibitor_clade_F_member_1
Elf1	0.69	0.008	E74-like_factor_1
Tnfrsf12a	0.69	0.03	tumor_necrosis_factor_receptor_superfamily_member_12a
Prrg1	0.70	0.014	proline_rich_Gla_(G-carboxyglutamic_acid)_1
C030034I22Rik	0.70	0.040	RIKEN_cDNA_C030034I22_gene
Nudt8	0.70	0.032	nudix_(nucleoside_diphosphate_linked_moiety_X)-type_motif_8
Cenpq	0.70	0.026	centromere_protein_Q
Gm37111	0.70	0.041	predicted_gene_37111

Gm14418	0.70	0.047	predicted_gene_14418
Grem1	0.71	0.020	gremlin_1_DAN_family_BMP_antagonist
Mcm3	0.71	0.044	minichromosome_maintenance_deficient_3_(S._cerevisiae)
Gm2007	0.72	0.041	predicted_gene_2007
1700024P16Rik	0.72	0.049	RIKEN_cDNA_1700024P16_gene
Il10ra	0.72	0.042	interleukin_10_receptor_alpha
Mcts2	0.72	0.019	malignant_T_cell_amplified_sequence_2
Pold4	0.73	0.023	polymerase_(DNA-directed)_delta_4
1190007I07Rik	0.73	0.043	RIKEN_cDNA_1190007I07_gene
4921539H07Rik	0.73	0.020	RIKEN_cDNA_4921539H07_gene
Ndp	0.73	0.036	Norrie_disease_(pseudoglioma)_(human)
Gm16867	0.73	0.047	predicted_gene_16867
Ube2l6	0.73	0.040	ubiquitin-conjugating_enzyme_E2L_6
Krt73	0.73	0.050	keratin_73
Rhobtb1	0.74	0.044	Rho-related_BTB_domain_containing_1
Gal3st1	0.74	0.011	galactose-3-O-sulfotransferase_1
Robo3	0.74	0.001	roundabout_homolog_3_(Drosophila)
Usb1	0.74	0.042	U6_snRNA_biogenesis_1
Isoc2b	0.75	0.039	isochorismatase_domain_containing_2b
Ica1	0.75	0.000	islet_cell_autoantigen_1
Gm21967	0.75	0.009	predicted_gene_21967
**Ifnar2**	**0.75**	**0.013**	**interferon_(alpha_and_beta)_receptor_2**
Zfp961	0.75	0.012	zinc_finger_protein_961
Trdmt1	0.75	0.044	tRNA_aspartic_acid_methyltransferase_1
Fcer1g	0.75	0.041	Fc_receptor_IgE_high_affinity_I_gamma_polypeptide
Lyn	0.75	0.031	Yamaguchi_sarcoma_viral_(v-yes-1)_oncogene_homolog
Gm43843	0.75	0.031	predicted_gene_43843

Kif26a	0.76	0.036	kinesin_family_member_26A
Matn4	0.76	0.021	matrilin_4
Rmdn1	0.77	0.035	regulator_of_microtubule_dynamics_1
Fam109a	0.77	0.030	family_with_sequence_similarity_109_member_A
Apln	0.77	0.007	apelin
Akr1b10	0.77	0.018	aldo-keto_reductase_family_1_member_B10_(aldose_reductase)
Lysmd1	0.77	0.038	LysM_putative_peptidoglycan-binding_domain_containing_1
Dnajc28	0.78	0.032	DnaJ_heat_shock_protein_family_(Hsp40)_member_C28
Lct	0.78	0.002	lactase
Gm15542	0.78	0.037	predicted_gene_15542
Dusp28	0.78	0.017	dual_specificity_phosphatase_28
Cbx8	0.78	0.039	chromobox_8
Gpt	0.79	0.031	glutamic_pyruvic_transaminase_soluble
Pon2	0.79	0.001	paraoxonase_2
Pot1a	0.79	0.048	protection_of_telomeres_1A
4930453N24Rik	0.79	0.007	RIKEN_cDNA_4930453N24_gene
Bbip1	0.80	0.010	BBSome_interacting_protein_1
Entpd1	0.80	0.047	ectonucleoside_triphosphate_diphosphohydrolase_1
Mobp	0.80	0.006	myelin-associated_oligodendrocytic_basic_protein
Fzd3	1.20	0.023	frizzled_class_receptor_3
Asns	1.20	0.000	asparagine_synthetase
Ctdspl2	1.20	0.028	CTD_(carboxy-terminal_domain_RNA_polymerase_II_polypeptide_A)_small_phosphatase_like_2
Zcchc12	1.20	0.014	zinc_finger_CCHC_domain_containing_12
Pisd-ps1	1.20	0.039	phosphatidylserine_decarboxylase_pseudogene_1
1110032A03Rik	1.21	0.017	RIKEN_cDNA_1110032A03_gene

Trmt112	1.21	0.020	tRNA_methyltransferase_11-2
Acot9	1.22	0.041	acyl-CoA_thioesterase_9
RP23-407N2.2	1.22	0.028	NA
Snx7	1.23	0.041	sorting_nexin_7
Mest	1.23	0.038	mesoderm_specific_transcript
Commd1	1.23	0.022	COMM_domain_containing_1
Arfgef3	1.23	0.000	ARFGEF_family_member_3
Unc5d	1.23	0.012	unc-5_homolog_D_(C._elegans)
Prkra	1.23	0.047	protein_kinase_interferon_inducible_double_stranded_RNA_dependent_activator
Dcn	1.23	0.035	decorin
Htra2	1.23	0.037	HtrA_serine_peptidase_2
Slc9a7	1.24	0.019	solute_carrier_family_9_(sodium/hydrogen_exchanger)_member_7
Usp35	1.24	0.011	ubiquitin_specific_peptidase_35
Chst8	1.25	0.019	carbohydrate_(N-acetylgalactosamine_4-0)_sulfotransferase_8
Rps4l	1.25	0.019	ribosomal_protein_S4-like
Sema4b	1.26	0.025	sema_domain_immunoglobulin_domain_(Ig)_transmembrane_domain_(TM)_and_short_cytoplasmic_domain_(semaphorin)_4B
Smo	1.26	0.015	smoothened_frizzled_class_receptor
Engase	1.26	0.037	endo-beta-N-acetylglucosaminidase
Thsd7b	1.26	0.031	thrombospondin_type_I_domain_containing_7B
Btbd11	1.27	0.015	kelch_repeat_and_BTB_(POZ)_domain_containing_11
Rnaset2b	1.27	0.027	ribonuclease_T2B
**Ppp2r3d**	**1.28**	**0.006**	**protein_phosphatase_2_(formerly_2A)_regulatory_subunit_B''_delta**
Cdyl	1.28	0.042	chromodomain_protein_Y_chromosome-like

Strip2	1.28	0.020	striatin_interacting_protein_2
Wdr6	1.29	0.000	WD_repeat_domain_6
Amd2	1.30	0.005	S-adenosylmethionine_decarboxylase_2
Rbm3	1.30	0.000	RNA_binding_motif_protein_3
Zfp830	1.30	0.025	zinc_finger_protein_830
C2cd4c	1.31	0.035	C2_calcium-dependent_domain_containing_4C
**Col6a1**	**1.31**	**0.008**	**collagen_type_VI_alpha_1**
Rbm11	1.31	0.038	RNA_binding_motif_protein_11
Npr3	1.32	0.003	natriuretic_peptide_receptor_3
Cirbp	1.33	0.000	cold_inducible_RNA_binding_protein
Pafah1b3	1.34	0.030	platelet-activating_factor_acetylhydrolase_isoform_1b_subunit_3
Grm8	1.34	0.043	glutamate_receptor_metabotropic_8
Zfp11	1.34	0.042	zinc_finger_protein_11
Slc7a3	1.35	0.038	solute_carrier_family_7_(cationic_amino_acid_transporter_y+_system)_member_3
Gm13292	1.35	0.029	predicted_gene_13292
Pdp2	1.35	0.032	pyruvate_dehyrogenase_phosphatase_catalytic_subunit_2
Scn7a	1.36	0.050	sodium_channel_voltage-gated_type_VII_alpha
Umad1	1.36	0.000	UMAP1-MVP12_associated_(UMA)_domain_containing_1
**Efna5**	**1.36**	**0.041**	**ephrin_A5**
Htatip2	1.37	0.022	HIV-1_Tat_interactive_protein_2
Klhl36	1.37	0.031	kelch-like_36
RP23-54G8.4	1.37	0.047	NA
Fam71e1	1.37	0.043	family_with_sequence_similarity_71_member_E1
Setmar	1.39	0.037	SET_domain_without_mariner_transposase_fusion
Pcdhgb4	1.39	0.046	protocadherin_gamma_subfamily_B_4

Kcnq4	1.39	0.024	potassium_voltage-gated_channel_subfamily_Q_member_4
Plcxd3	1.41	0.026	phosphatidylinositol-specific_phospholipase_C_X_domain_containing_3
Xaf1	1.41	0.040	XIAP_associated_factor_1
Tpbg	1.41	0.031	trophoblast_glycoprotein
Rom1	1.42	0.024	rod_outer_segment_membrane_protein_1
Pcdhb15	1.42	0.043	protocadherin_beta_15
Kcnh5	1.42	0.049	potassium_voltage-gated_channel_subfamily_H_(eag-related)_member_5
Gm9531	1.43	0.048	predicted_gene_9531
Gm9987	1.43	0.040	predicted_gene_9987
Akna	1.43	0.018	AT-hook_transcription_factor
Crb2	1.43	0.036	crumbs_family_member_2
Gm37874	1.43	0.036	predicted_gene_37874
Kcnu1	1.44	0.035	potassium_channel_subfamily_U_member_1
Loxl1	1.45	0.030	lysyl_oxidase-like_1
Dnph1	1.45	0.032	2'-deoxynucleoside_5'-phosphate_N-hydrolase_1
Fam69c	1.45	0.013	family_with_sequence_similarity_69_member_C
Rps10-ps2	1.45	0.027	ribosomal_protein_S10_pseudogene_2
Krt9	1.45	0.001	keratin_9
Gm37034	1.47	0.032	predicted_gene_37034
Abcb4	1.47	0.008	ATP-binding_cassette_sub-family_B_(MDR/TAP)_member_4
Tha1	1.47	0.021	threonine_aldolase_1
Hfm1	1.47	0.046	HFM1_ATP-dependent_DNA_helicase_homolog
C1qtnf6	1.48	0.036	C1q_and_tumor_necrosis_factor_related_protein_6
Gcnt1	1.48	0.026	glucosaminyl_(N-acetyl)_transferase_1_core_2
4632427E13Rik	1.48	0.021	RIKEN_cDNA_4632427E13_gene

Aim2	1.48	0.025	absent_in_melanoma_2
Pcdhga8	1.48	0.002	protocadherin_gamma_subfamily_A_8
Fdxacb1	1.48	0.029	ferredoxin-fold_anticodon_binding_domain_containing_1
Adamts15	1.48	0.031	a_disintegrin-like_and_metallopeptidase_(reprolysin_type)_with_thrombospondin_type_1_motif_15
Kcne1l	1.49	0.020	potassium_voltage-gated_channel_Isk-related_family_member_1-like_pseudogene
Spag5	1.49	0.008	sperm_associated_antigen_5
Gm9762	1.50	0.020	predicted_pseudogene_9762
Tfr2	1.51	0.017	transferrin_receptor_2
**Col4a5**	**1.51**	**0.019**	**collagen_type_IV_alpha_5**
Pcdhgb7	1.52	0.009	protocadherin_gamma_subfamily_B_7
**Met**	**1.55**	**0.012**	**met_proto-oncogene**
Baiap3	1.55	0.020	BAI1-associated_protein_3
Gm12183	1.56	0.011	predicted_gene_12183
Gm14964	1.56	0.010	predicted_gene_14964
Bace2	1.57	0.013	beta-site_APP-cleaving_enzyme_2
Scnn1a	1.57	0.002	sodium_channel_nonvoltage-gated_1_alpha
Pcdhb5	1.58	0.008	protocadherin_beta_5
Pygl	1.61	0.003	liver_glycogen_phosphorylase
Pdk4	1.64	0.007	pyruvate_dehydrogenase_kinase_isoenzyme_4
Htra3	1.75	0.001	HtrA_serine_peptidase_3

*The genes in the PI3K-Akt signaling pathway were highlighted.

Supplementary Figure 1d

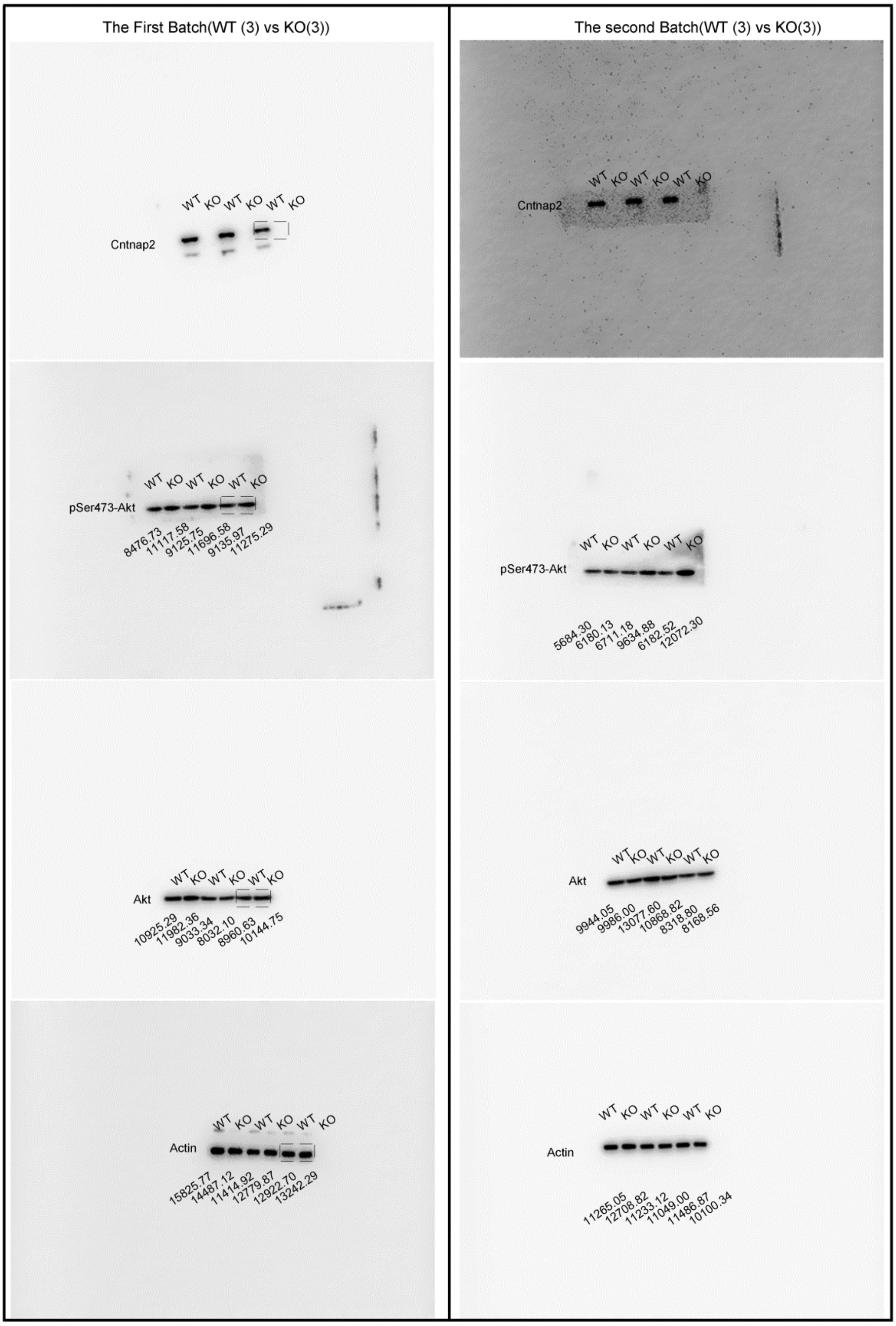

Fig. 1d. The uncropped Western blot images of Cntnap2, pSer473-Akt, Akt and Actin from the hippocampus of 6 WT and 6 Cntnap2-/- mice. The boxed images were shown in Figure 1d. The band density was quantified using ImageJ software, and the

scores were listed at the bottom of respective bands.

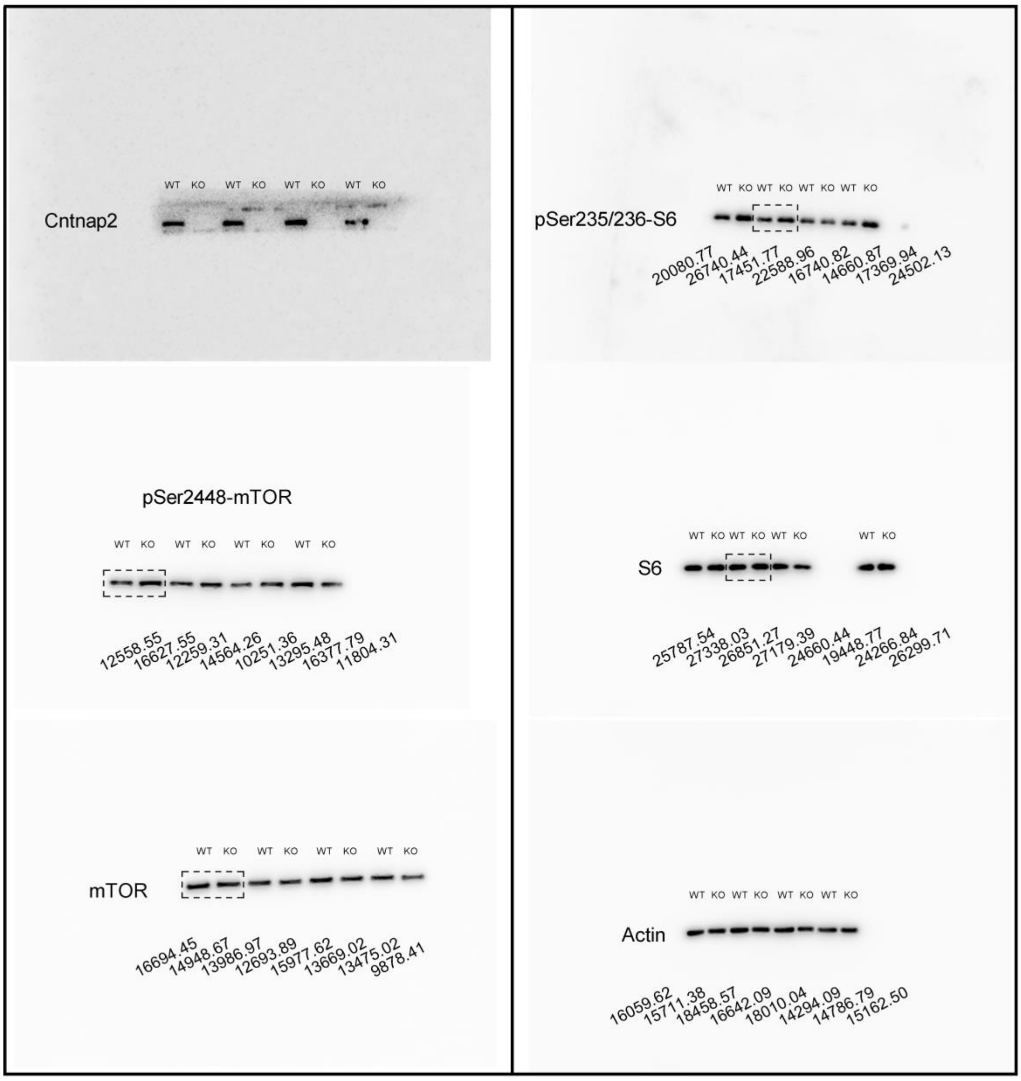

Fig. 1d. The uncropped Western blot images of Cntnap2, pSer2448-mTOR, mTOR, pSer235/236-S6, S6 and Actin from the hippocampus of 4 WT and 4 Cntnap2-/- mice. The boxed images were shown in Figure 1d. The band density was quantified using ImageJ software, and the scores were listed at the bottom of respective bands.

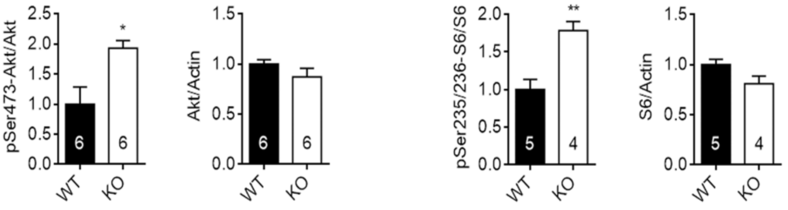

Fig. 1d. Quantification of lysates from the cortex of WT and *Cntnap2-/-* mice in

Akt-mTOR signaling pathway. *$p < 0.05$, **$p < 0.01$. Data are expressed as the mean ± sem, unpaired two-tail Student's t test. The uncropped Western blot images of Cntnap2, pSer473-Akt, Akt, pSer235/236-S6, S6 and Actin were provided below.

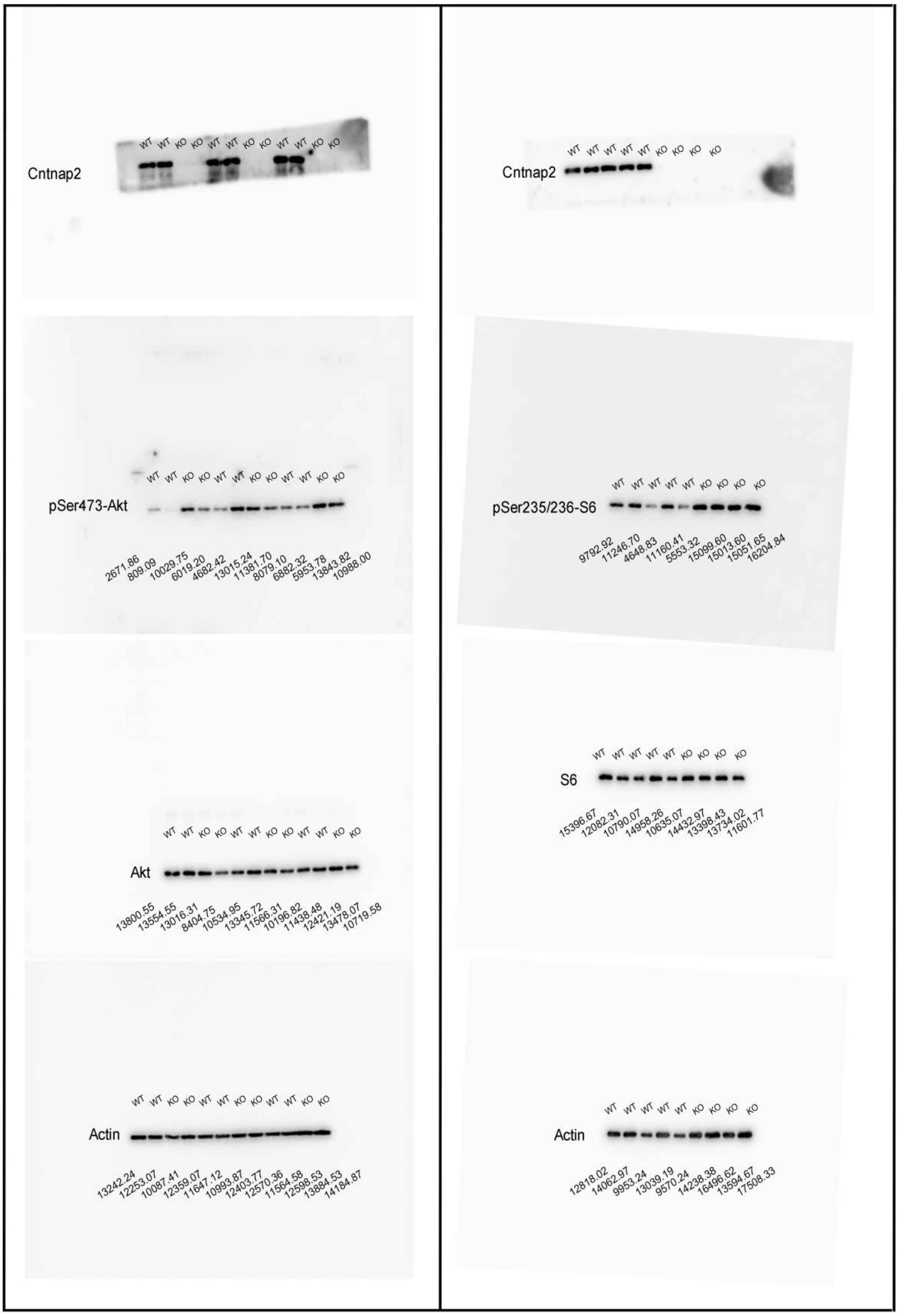

Fig. 1d. The uncropped Western blot images of Cntnap2, pSer473-Akt, Akt, pSer235/236-S6, S6 and Actin from the cortex of WT and Cntnap2-/- mice. 6 WT and 6 Cntnap2-/- mice for Cntnap2, pSer473-Akt, Akt and Actin. 5 WT and 4 Cntnap2-/- mice for Cntnap2, pSer235/236-S6, S6 and Actin. The band density was quantified using ImageJ software, and the scores were listed at the bottom of respective bands.

Supplementary Figure. 2b

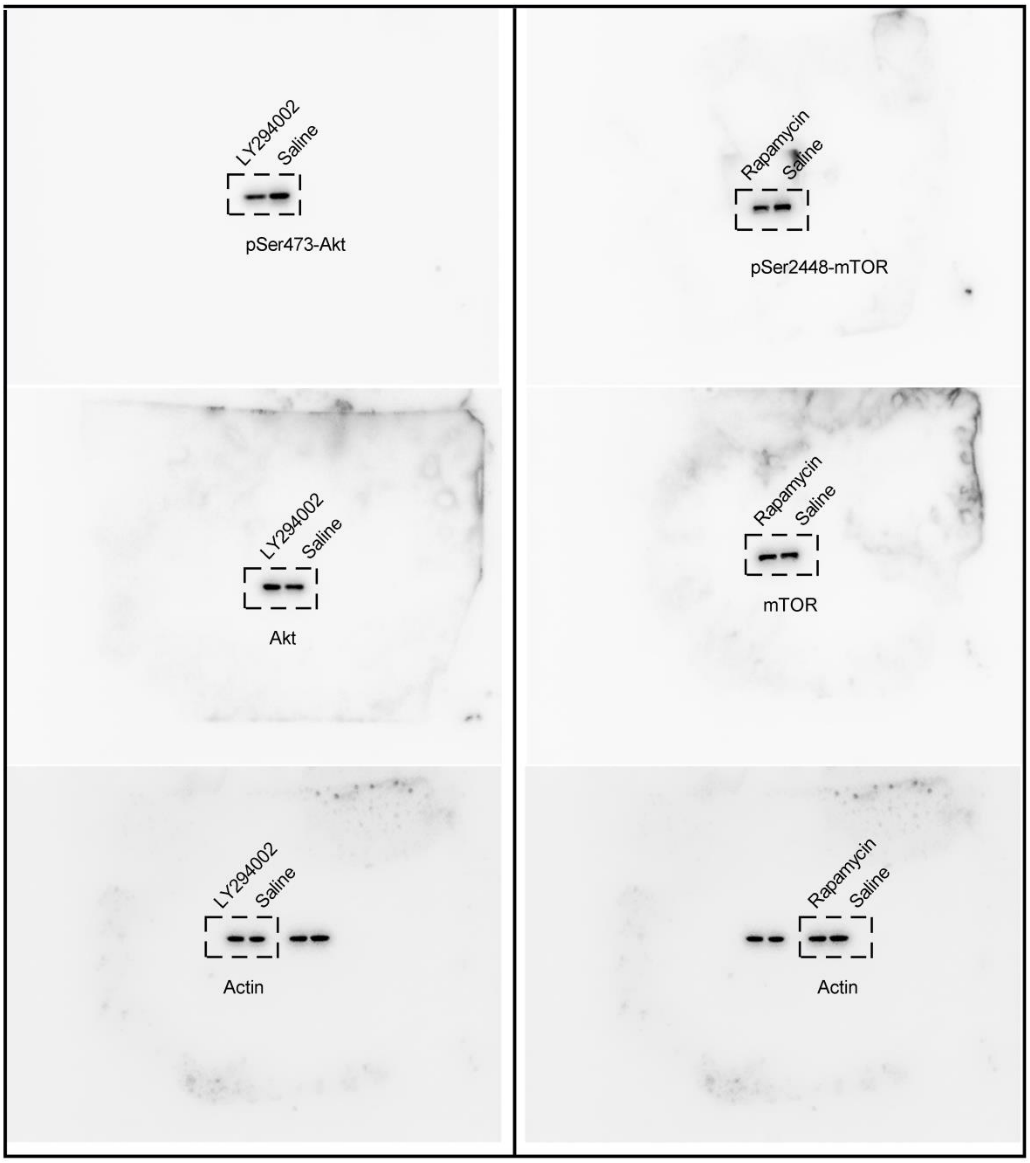

Fig. 2b The uncropped Western blot images of pSer473-Akt, Akt, pSer2448-mTOR, mTOR and Actin from the hippocampus of WT mice after 60 min of the second

injection with LY294002 or rapamycin respectively. The boxed images were shown in Figure 2b.
